# Pseudoarthrosis after anterior cervical discectomy and fusion: rate of occult infections and outcome of anterior revision surgery

**DOI:** 10.1186/s12891-023-06819-7

**Published:** 2023-08-29

**Authors:** Anna-Katharina Calek, Elin Winkler, Mazda Farshad, José Miguel Spirig

**Affiliations:** 1 Department of Orthopedics, Balgrist University Hospital, University of Zurich, Forchstrasse 340, Zurich, CH-8008 Switzerland; 2 Balgrist University Hospital, University Spine Center Zurich, University of Zurich, Zurich, Switzerland

**Keywords:** Pseudoarthrosis, Revision surgery, Anterior cervical discectomy and fusion, ACDF, Cervical spine

## Abstract

**Background:**

Pseudoarthrosis after anterior cervical discectomy and fusion (ACDF) is relatively common and can result in revision surgery. The aim of the study was to analyze the outcome of patients who underwent anterior revision surgery for pseudoarthrosis after ACDF.

**Methods:**

From 99 patients with cervical revision surgery, ten patients (median age: 48, range 37–74; female: 5, male: 5) who underwent anterior revision surgery for pseudoarthrosis after ACDF with a minimal follow up of one year were included in the study. Microbiological investigations were performed in all patients. Computed tomography (CT) scans were used to evaluate the radiological success of revision surgery one year postoperatively. Clinical outcome was quantified with the Neck Disability Index (NDI), the Visual Analog Scale (VAS) for neck and arm pain, and the North American Spine Society Patient Satisfaction Scale (NASS) 12 months (12–60) after index ACDF surgery. The achievement of the minimum clinically important difference (MCID) one year postoperatively was documented.

**Results:**

Occult infection was present in 40% of patients. Fusion was achieved in 80%. The median NDI was the same one year postoperatively as preoperatively (median 23.5 (range 5–41) versus 23.5 (7–40)), respectively. The MCID for the NDI was achieved 30%. VAS-neck pain was reduced by a median of 1.5 points one year postoperatively from 8 (3–8) to 6.5 (1–8); the MCID for VAS-neck pain was achieved in only 10%. Median VAS-arm pain increased slightly to 3.5 (0–8) one year postoperatively compared with the preoperative value of 1 (0–6); the MCID for VAS-arm pain was achieved in 14%. The NASS patient satisfaction scale could identify 20% of responders, all other patients failed to reach the expected benefit from anterior ACDF revision surgery. 60% of patients would undergo the revision surgery again in retrospect.

**Conclusion:**

Occult infections occur in 40% of patients who undergo anterior revision surgery for ACDF pseudoarthrosis. Albeit in a small cohort of patients, this study shows that anterior revision surgery may not result in relevant clinical improvements for patients, despite achieving fusion in 80% of cases.

**Level of evidence:**

Retrospective study, level III.

## Introduction

The first description of anterior cervical discectomy and fusion (ACDF), also known as Smith-Robinson procedure, dates back to the 1950s [1]. Since then, ACDF has been successfully used to treat cervical degenerative diseases, as well as cervical spine injuries [2, 3]. The aim of the procedure is to treat neck pain, radiculopathy, and myelopathy by adequate decompression and rapid fusion. Pseudoarthrosis and adjacent segment degeneration are known complications of this procedure and may result in persistent symptoms requiring revision surgery [3–5]. A nonunion rate between 0 and 20% for single level fusions is reported in the literature [6, 7]. In addition to patient risk factors such as older age, diabetes, and smoking [8–10], several surgical factors such as multilevel fusions, choice of instrumentation, and bone grafting may influence the postoperative results ^6–8^. Pseudoarthrosis after ACDF can be addressed using an anterior or posterior approach [6, 11–13]. Revision through a posterior approach provides new biology for fusion and avoids scarring from the index procedure, which can hamper dissection and potentially cause serious complications such as vertebral artery injury and esophageal or tracheal perforation [14]. However, the posterior approach requires excessive dissection of the extensor muscles from their bony attachments, resulting in extensive soft tissue injury [15], which is associated with a higher rate of wound healing problems and deep wound infections [16–18]. In addition, higher perioperative blood loss and longer postoperative recovery time have been reported compared to the anterior approach [15]. Furthermore, some complications, such as graft migration or cervical kyphosis, sometimes cannot be treated from posterior.

In general, revision surgery is associated with longer hospital stays, higher costs, and increased morbidity [19]. Nevertheless, satisfactory clinical and radiological outcomes have been reported after posterior revision surgery for pseudoarthrosis after ACDF [19, 20]. To date, it is unclear whether cervical pseudoarthroses should be treated from posterior or anterior. The aim of the study was to analyze the outcome of patients who underwent anterior revision surgery for pseudoarthrosis after ACDF.

## Materials and methods

**Fig. 1:**
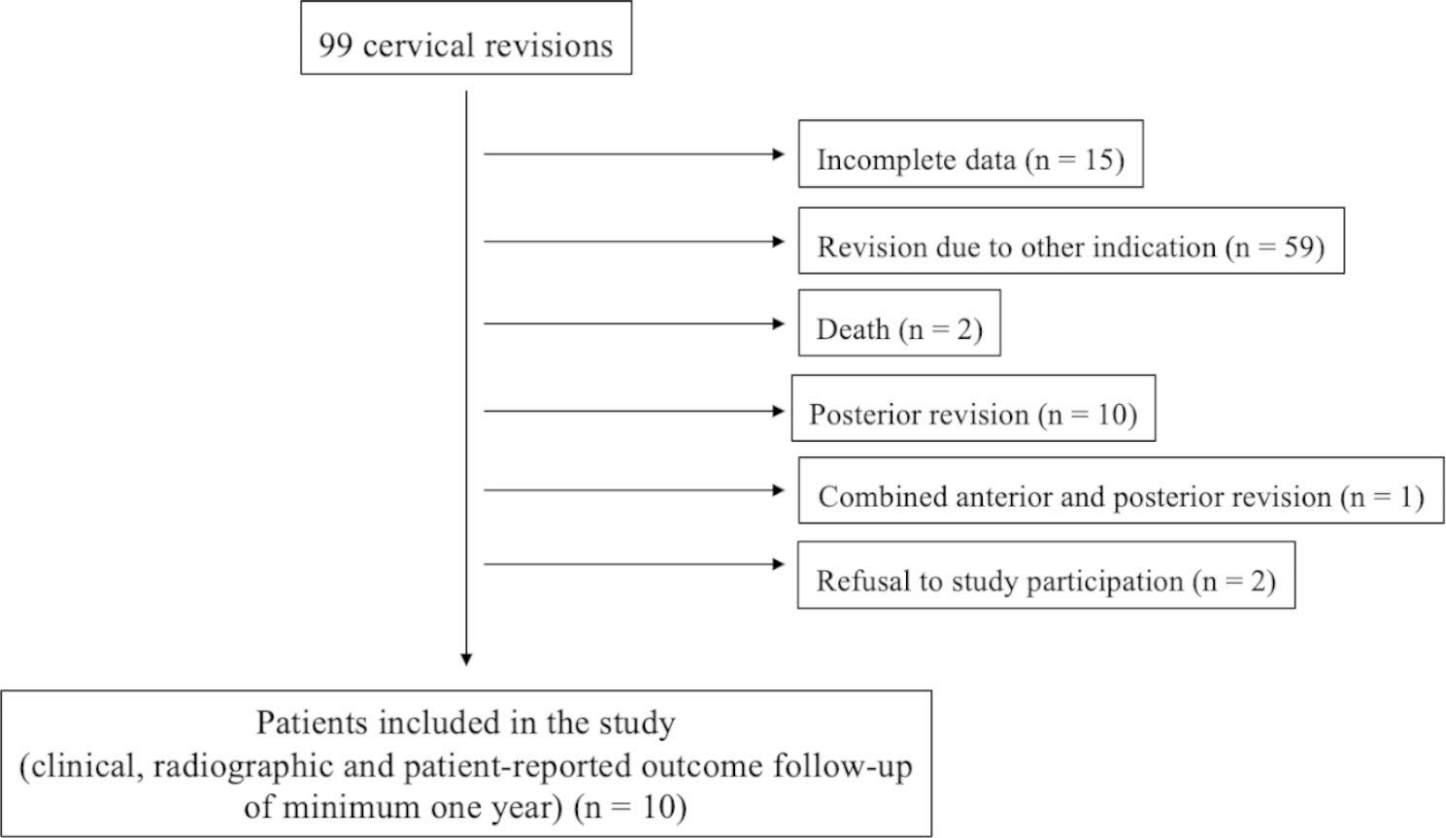
Patient’s flowchart

The study was approved by the responsible investigational review board (KEK-ZH-Nr. 2022 − 00575) and conducted following the Helsinki Declaration. Clinical and radiographic data of patients who underwent anterior revision surgery for pseudoarthrosis after ACDF between 2017 and 2021 were analyzed in a retrospective fashion. Pseudoarthrosis was defined as the absence of bridging bone across the fused levels on CT scans [5]. Ten patients with available pre- and postoperative clinical outcome scores and radiological follow-up of at least one year were included in this period (Fig. 1). Patients with incomplete follow-up data were excluded. Patient demographics as well as the indication for the index surgery are listed in Table 1. An anterior plate was used at the index surgery in 50% of the cases. All patients evaluated in this study had complaints of axial neck pain, and eight patients also noted a radiculopathy manifested by arm pain, paresthesia, or weakness of the muscles of the affected nerve root.

**Table 1:** Demographics and indications for anterior cervical discectomy and fusion (index procedure)

Age (years)^§^	48 (37–74)
Weight^§^	80.6 kg (63-113.7)
Height^§^	167.5 cm (155–183)
Smoker	yes: 4; no: 6
Myelopathy	2
Radiculopathy	3
Spinal stenosis	1
Cervicobrachialgia	4

^§^ Values in median and ranges ()

### Surgical technique

In all cases, the pre-existing scar was used to access the affected vertebrae. The sternocleidomastoid muscle was brought laterally. Then, the cervical spine was exposed by dissecting the interval between the neurovascular bundle laterally and the esophagus and trachea medially. The foreign material (plate (in five cases), cage) was visualized and removed. All foreign material was sent to the microbiologic laboratory for microbiological evaluation. Furthermore, superficial and deep samples (six in total) were obtained from the surgical site for microbiological workup. Care was taken to ensure that these did not come into contact with the patient’s skin in order to avoid contamination. The pseudoarthrosis was refreshed, and the foramina were re-decompressed as needed. A new cage, previously filled with iliac crest autograft, was then inserted. A ventral plate was used to enhance rigidity of the construct. In one case, a corpectomy of a vertebra between two adjacent pseudoarthrosis was performed.

### Clinical outcome

Postoperative clinical assessment was performed in an institutionally standardized manner at follow-up intervals of 4 weeks, 6 months, and then annually. Clinical examination included assessment of the neck disability index (NDI) [21], visual analog scale (VAS) for neck and arm pain, and the North American Spine Society (NASS) patient satisfaction scale [22]. The achievement of the minimum clinically important difference (MCID) one year postoperatively for the NDI, VAS-neck pain, and VAS-arm pain was assessed. We followed the definition of the MCID by Parker et al. [23] for the NDI, VAS-neck pain, and VAS-arm pain as -17.3%, -2.6 points, and − 4.1 points, respectively. Satisfaction was assessed based on the NASS patient satisfaction scale analyzed one year postoperatively. The NASS patient satisfaction scale has previously been used as an anchor for the definition of the MCID [23]. This 4-item questionnaire indicates the patient’s postoperative satisfaction: (1) “The treatment met my expectations”; (2) “I did not improve as much as I had hoped, but I would undergo the same treatment for the same outcome”; (3) “I did not improve as much as I had hoped, and I would not undergo the same treatment for the same outcome”; and (4) “I am the same or worse than before the treatment.” For the purposes of this study, patients who answered choice “the treatment met my expectations” were considered as responders, all other answers were classified as non-responders [23].

### Radiographic outcome

One year postoperatively, a spiral 128-slice multidetector CT image (SOMATOM Edge Plus, Siemens Healthcare GmbH, Erlangen, Germany) with a slice thickness of < 1 mm was obtained from all revised cervical spines. Detection of a solid bone bridge on CT scan one year postoperatively was defined as radiological success of revision surgery. Because of the higher sensitivity and specificity for assessing the internal and external bone bridge compared with conventional radiographs or dynamic flexion-extension radiographs [5], the success of the revision procedure was evaluated primarily on the basis of postoperative CT scans in the sagittal, transverse, and coronal planes using Merlin 5.2. (Phoenix-PACS, Freiburg, Germany) by a board-certified musculoskeletal radiologist and a fellowship-trained orthopaedic surgeon.

Furthermore, operative time, radiographic exposure, blood loss, and hospitalization time were analyzed.

### Statistical analysis

Statistical analysis was conducted with SPSS software v27.0 (IBM, New York, USA). Statistics were limited to descriptive analysis due to the relatively small sample size (as a result of a rare event). For continuous variables, the medians are given together with their ranges.

## Results

Four patients developed pseudoarthrosis after single-level ACDF, four patients after two-level ACDF, and two patients after three-level ACDF. Pre- and postoperative clinical and radiographic outcome parameters are presented in Table 2. Clinical outcome measures at one year postoperatively in patients with successful fusion after revision surgery and in patients with persistent pseudoarthrosis are presented in Table 3. The median clinical and radiographic follow-up was 19 months (range: 12–36 months). The median time from index surgery to revision surgery was 26.5 months (range: 7–84 months).

**Table 2:** Clinical and radiographic outcome

Clinical outcome	
**NDI** Preoperative One year postoperatively Achievement of MCID	23.5 (7–40) 23.5 (5–41) yes: 3; no: 7
**VAS-neck pain** Preoperative One year postoperatively Achievement of MCID	8 (3–8) 6.5 (1–8) yes: 1; no: 9
VAS-arm pain* Preoperative One year postoperatively Achievement of MCID	1 (0–6) 3.5 (0–8) yes: 1; no: 7
**NASS** Preoperative One year postoperatively	- responder: 2; non-responder: 8^§^
**Radiographic outcome**	
Verified bony fusion	yes: 8; no: 2

Values in median and ranges ()

NDI: Neck Disability Index, VAS: visual analog scale, NASS: American Spine Society patient satisfaction scale

MCID: minimum clinically important difference

^*^ VAS-arm pain was assessed in patients with preoperative radiculopathy or cervicobrachialgia (n = 8)

^§^ of the non-responders, three patients hoped for greater improvement, but would undergo the revision surgery again (answer 2); two patients would not undergo the revision surgery again (answer 3); and one patient reported worse symptoms postoperatively (answer 4)

**Table 3:** Clinical Outcome Measures at one year postoperatively in patients with successful revision surgery and patients with persistent pseudoarthrosis

	NDI	MCID	VAS-neck pain	MCID	VAS-arm pain	MCID	NASS	Responder	Union
**Patient 1**	10	no	6	no	0	-	2	no	yes
**Patient 2**	22	no	6	no	0	no	3	no	yes
**Patient 3**	33	no	8	no	5	-	4	no	yes
**Patient 4**	25	no	8	no	8	no	2	no	yes
**Patient 5**	5	yes	4	no	0	no	1	yes	yes
**Patient 6**	41	no	8	no	4	no	2	no	yes
**Patient 7**	10	yes	6	no	7	no	1	yes	yes
**Patient 8**	34	no	7	no	6	no	3	no	yes
**Patient 9**	30	no	8	no	3	no	3	no	no
**Patient 10**	5	yes	1	yes	1	yes	2	no	no

The microbiological workup was able to detect a low-grad infection in four cases (40%). The pathogen was Cutibacterium acnes in all cases. In three patients, the pathogen was found on the foreign material and in the deep tissue samples; in one patient it was found in the deep tissue and bone samples. These patients were treated with antibiotics for six weeks.

### Low-grade infections

Occult infection was confirmed in 40% (in 4/10 patients; at least 3/6 samples harvested were positive). Cutibacterium acnes was detected in all cases. In three out of four patients, the infection was successfully treated - bony healing was achieved. Clinical outcome one year postoperatively did not differ between patients with low-grade infection and those without infection.

### Clinical outcome

The median NDI was the same one year postoperatively as preoperatively (Table 2). The MCID for the NDI was achieved in three out of ten cases. VAS-neck pain was reduced by a median of 1.5 points one year postoperatively, and the MCID for VAS-neck pain was achieved in only one of ten cases with neck pain. The median VAS-arm pain increased slightly to 3.5 (0–8) one year postoperatively compared with the preoperative value of 1 (0–6); the MCID for VAS-arm pain was achieved in one of seven patients (14.3%) with arm pain. The NASS patient satisfaction scale could identify two responders, all other patients did not benefit from anterior revision surgery by definition [23] . However, six out of ten patients (60%) would undergo the revision surgery again in retrospect.

### Radiographic outcome

**Fig. 2:**
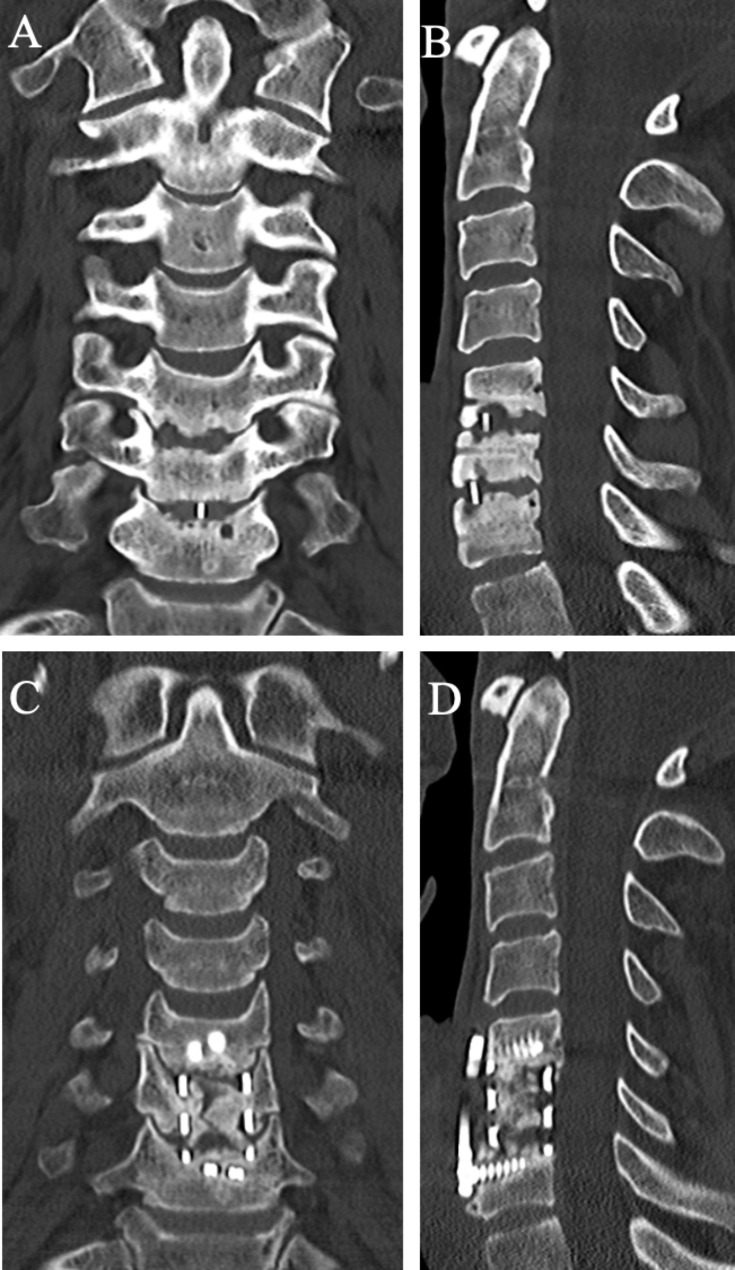
Postoperative coronal and sagittal CT two years after the index surgery shows pseudoarthrosis at C5/6 and C6/7 (A + B). Coronal and sagittal postoperative CT one year after revision surgery shows persistent pseudoarthrosis (D + E)

In eight patients (80%), fusion was successfully achieved. Persistent pseudoarthrosis could be detected in two patients (20%). The first case was a 40-year-old-male who developed pseudoarthrosis after ACDF C5/6 and C6/7. Due to nuchalgias, the patient underwent an anterior revision. One and a half years later, the patient was symptom-free and satisfied with the result, but the CT scan showed pseudoarthrosis again (Fig. 2). No further intervention was necessary as the patient was clinically asymptomatic. The second case was a 58-year-old male who had undergone multiple prior surgeries and developed C3/4 and C6/7 pseudoarthrosis after a multilevel ACDF. Anterior revision surgery was performed, and cutibacterium acnes was cultivated in the intraoperative samples. The patient subsequently received a postoperative antibiotic therapy. One and a half years postoperatively, CT imaging showed a fusion of the segments C3/4, however, the pseudoarthrosis C6/7 was still present.

### Operative time, radiographic exposure, blood loss, and hospitalization time

The median operative time for the revision surgery was 159,5 minutes (range: 90 to 260 minutes) with a median blood loss of 150 ml (range: 50 to 300 ml). The median intraoperative fluoroscopic dose was 103.5 mGycm^2^ (range: 19.1 to 264.5 mGycm^2^). The median length of hospital stay was 5.5 days (range: 4 to 8 days).

### Illustrative case 1 (Fig. 2): satisfactory clinical result despite failed revision

A patient underwent an ACDF C5/6 and C6/7 in 2018. Unfortunately, fusion of the segments did not occur, resulting in pseudoarthrosis and nuchalgia, leading to revision surgery from anterior with corpectomy, autologous bone grafting from the iliac crest, and ventral fusion two years later. Fifteen months postoperatively, the patient was asymptomatic, but the pseudoarthrosis persisted. Preoperative coronal (A) and sagittal (B) CT scan shows pseudoarthrosis after ACDF C5/6 and C6/7. Postoperative coronal (C) and sagittal (D) CT scan showing persisting pseudoarthrosis after corpectomy, autologous bone grafting, and ventral fusion.

### Illustrative case 2 (Fig. 3): unsatisfactory result despite successful revision

**Fig. 3:**
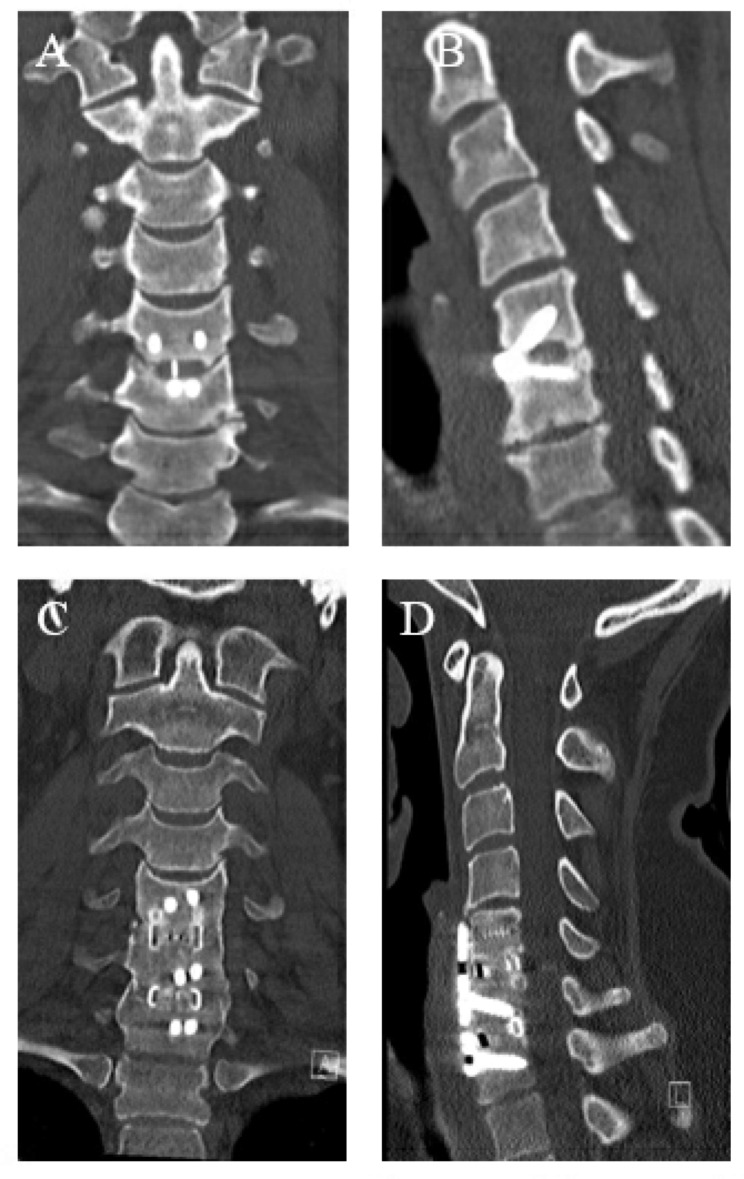
Coronal and sagittal postoperative CT 5.5 years after the index procedure shows pseudoarthrosis C5/6 (A + B). Coronal and sagittal postoperative CT after revision with satisfactory fusion (D + E)

The patient underwent ACDF C5/6 for a paramedian disc herniation. Follow-up revealed a pseudoarthrosis at C5/6 and an adjacent segment degeneration at C6/7 were detected. Due to persistent bilateral cervicobrachialgia, revision surgery was performed 5.5 years later. The revision surgery consisted of removal of the material, insertion of cages filled with autograft from the iliac crest, and ventral fusion of C5/6 and C6/7. Two and a half years after the revision surgery, the fusion was satisfactory, but the patient continued to suffer from severe neck pain and could not subjectively benefit from the surgery. Preoperative coronal (A) and sagittal (B) CT scans show pseudoarthrosis after ACDF C5/6. Postoperative coronal (C) and sagittal (D) CT scans show satisfactory fusion.

## Discussion

The present study specifically looked at the outcomes of *anterior* revision surgery for pseudoarthrosis after ACDF. The main findings of our study are that (1) occult infection was detected in 40% of ACDF pseudoarthroses without prior suspicion of infection and that (2) anterior revision surgery, although achieving an 80% fusion rate, did not result in relevant clinical improvement in the majority of patients.

Pseudoarthrosis after ACDF may be asymptomatic but may also compromise the clinical outcome [24, 25]. The incidence of pseudoarthrosis after ACDF varies widely in the literature [24]. This is mainly explained by diverging definitions of pseudoarthrosis, the lack of standardized radiographic criteria [5], the type of bone graft used (with a reported mean pseudoarthrosis rate of 4.8% in allograft studies, and 0.9% in autograft studies [26]), the number of levels fused, as well as patient-specific risk factors such as smoking [8], follow-up time and the surgical of approach [15]. Symptomatic pseudoarthrosis after ACDF is usually treated by revision surgery, by an anterior, posterior or a combined approach. Each approach has its advantages and disadvantages: The posterior approach avoids dissection through scar tissue with the associated risk of injuring the laryngeal nerve [27], the carotid and vertebral arteries, trachea and esophagus [28]. However, it is associated with higher wound complication rates due to disruption of the posterior musculature, higher perioperative blood loss and longer postoperative recovery time [15]. The anterior approach better addresses graft migration, cervical kyphosis or suspected infection of the implants. The latter should not be underestimated: The most important unexpected finding was the high rate of low-grade infections (40%). While pseudoarthrosis is commonly associated with low-virulence bacteria, to the best of our knowledge, there have been no studies of occult infections in revision surgery for cervical pseudoarthrosis [29], so no comparative values can be used. Burkhard et al. [30] reported a 10% rate of occult infection in patients undergoing thoracolumbar pseudoarthrosis revision after spinal fusion without preoperative clinical suspicion. In the present study, the rate was four times higher. Reasons or patient-specific risk factors like body mass index or diabetes mellitus could not be identified because of the small number of cases. Nevertheless, occult infection should be considered in the absence of bony healing, and a routine microbiologic sampling should be done during revision of cervical spinal pseudoarthrosis.

The aforementioned unique advantages of the anterior approach for revision of ACDF pseudoarthrosis make it a beneficial procedure, although fusion rates are reported to be higher with the posterior approach. Studies of posterior repair of pseudoarthrosis have reported fusion rates of 94 to 100% [6, 20, 25], but with methodological limitations [5]. Carreon et al. [15] compared outcomes after posterior and anterior revision surgery and concluded that posterior fusion was more successful than anterior fusion in treating pseudoarthrosis: second revision surgery for persistent nonunion was necessary considerably more often after an anterior revision procedure (44% versus 2.2%; need for second revision after anterior revision versus posterior revision); however, the complication rate (wound infection, bone graft site infection) was twice as high in the posterior group.

Furthermore, some studies have reported the anterior approach as an excellent revision strategy, with fusion rates ranging from 81 to 100% [12, 13, 31]. In the present study, the fusion rate was 80%. One reason that the fusion rate described here is at the lower end of the previously reported ranges could be the assessment of fusion with a CT scan, which can visualize pseudoarthrosis with high sensitivity [5].

In contrast to our results, the existing literature indicates that patients are overall satisfied and benefit from revision ACDF surgery: after posterior revision, patient satisfaction rates between 72 and 88% have been reported [6, 19, 20] and after anterior revision, these rates are slightly lower but still satisfactory (59–86%) [6, 13, 20]. In this study, only one patient reached the MCID in all clinical scores assessed (NDI, VAS-neck pain, VAS-arm pain, NASS) despite an overall 80% fusion success rate. Interestingly, this exact patient had persistent but asymptomatic pseudoarthrosis. Reasons for the poorer outcome described in the cohort may be that the clinical outcome in the above-mentioned studies was not assessed with the same scores including MCID [6, 13, 20] or that the definition of a “responder” and “non-responder” using the NASS score was more generous towards responders [19]. Therefore, the reports of “satisfaction” cannot be compared directly between available studies. Overall, this study looked at a small cohort, therefore the results should be interpreted with caution.

The following methodological limitations should be considered when interpreting and comparing the results: The small sample size (as a result of a relatively rare event) could introduce bias that can only be addressed by larger studies comparing both approaches in a randomized fashion. However, we believe that the results will allow better patient counseling and support surgical decision making. Because of the high rate of occult low-grade infection, we advocate the collection of multiple deep tissue samples for microbiological examination and routine submission of the removed hardware for sonication in all cervical pseudoarthrosis revisions. Otherwise, occult infections cannot be treated with targeted antibiotics, and symptoms may persist.

Based on the results of the present study, however, it is also evident that successful fusion after revision surgery is not necessarily associated with a better postoperative outcome. Therefore, patients should be educated about the controversial clinical benefits of revision surgery and the possibility of residual symptoms.

## Conclusion

Occult infections occur in 40% of patients who undergo anterior revision surgery for ACDF pseudoarthrosis. Albeit in a small cohort of patients, this study shows that anterior revision surgery may not result in relevant clinical improvements for patients, despite achieving fusion in 80% of cases.

## Data Availability

the dataset used and/or analysed during the current study available from the corresponding author on reasonable request.
